# Strand bias structure in mouse DNA gives a glimpse of how chromatin structure affects gene expression

**DOI:** 10.1186/1471-2164-9-16

**Published:** 2008-01-14

**Authors:** Kenneth J Evans

**Affiliations:** 1 School of Crystallography, Birkbeck College, University of London, Malet Street, London, WC1E 7HX, UK

## Abstract

**Background:**

On a single strand of genomic DNA the number of As is usually about equal to the number of Ts (and similarly for Gs and Cs), but deviations have been noted for transcribed regions and origins of replication.

**Results:**

The mouse genome is shown to have a segmented structure defined by strand bias. Transcription is known to cause a strand bias and numerous analyses are presented to show that the strand bias in question is not caused by transcription. However, these strand bias segments influence the position of genes and their unspliced length. The position of genes within the strand bias structure affects the probability that a gene is switched on and its expression level. Transcription has a highly directional flow within this structure and the peak volume of transcription is around 20 kb from the A-rich/T-rich segment boundary on the T-rich side, directed away from the boundary. The A-rich/T-rich boundaries are SATB1 binding regions, whereas the T-rich/A-rich boundary regions are not.

**Conclusion:**

The direct cause of the strand bias structure may be DNA replication. The strand bias segments represent a further biological feature, the chromatin structure, which in turn influences the ease of transcription.

## Background

Because of the Watson-Crick structure of DNA – A paired with T and C with G – the number of As must equal the number of Ts when the bases on both strands are counted. Although this equality does not have to be true for a single strand, Chargaff's second law refers to the equality of A/T and C/G bases on a single strand [1] and broadly speaking eukaryote genomes are free of intrastrand bias [2].

Early work on strand bias analysed prokaryote and viral genomes where strand biases have been observed and associated with origins of replication: the leading strand is found to be G-rich and T-rich, with the G-C bias often being found to be more consistent than the A-T bias [3-6].

Strand bias has been discovered at transcription start sites in plants and fungi [7], animals [8,9], and splice sites [10]. Strand bias has been found for long regions of DNA around actual and putative origins of replication [11]. An analysis of nearby divergent genes concluded that both replication and transcription effects were important for strand bias in a range of eukaryotes [12], a result confirmed by an analysis of the bias in large vertebrate genes [13]. Strand bias for transcribed regions has been ascribed to transcription coupled repair [14], but some categories of SNPs do not follow the pattern [15]. There is a weak (~0.3) correlation between expression of human genes and strand bias [16]. In human genes, the strand bias has been shown to be confined to non-coding regions and accentuated at boundary regions [17]. By reversing the argument, strand bias can be used to find transcribed regions [18]: this method predicts many more transcribed regions.

This paper has some similarities with a very recent paper by Huvet *et al*. [19] which also finds that the number of genes and their expression is more abundant near origins of replication (identified by strand bias markers), with transcription and replication usually being in the same direction. However, their domains are much larger than our segments. They use data on replication timing to support the interpretation of DNA replication: this paper uses an H-rule analysis to support the interpretation of chromatin organisation. Their mathematical model is to look for "N-shaped" skew patterns: ours is to look for segments of alternating bias. Hence they do not find an equivalent to the T-rich/A-rich boundary.

The present work has its origins in a number of peculiarities in the data. Firstly, the strand bias around the transcription start site is highly variable; secondly, an average bias can be seen in the data for hundreds of thousands of bases upstream and downstream of the start site; thirdly, in a large random piece of DNA, say 500 kb bases, (whether or not from a transcribed region), there is a large negative correlation between (A-T) and (G-C) [20]. These results can occur because there are long-range correlations between bases [21]. These peculiarities have led to the hypothesis that the genome is composed of strand bias segments and this paper demonstrates this. Given that there is an effect whereby transcription causes strand bias, there is a burden of proof to discharge to show that there is also a line of causality from strand bias to the placement of genes and their expression: we therefore give numerous arguments to make this point.

Although this paper emphasises that the strand bias discussed here is not caused by transcription, the main result is that there is a strand bias structure to the genome and this structure affects the placement of genes and the probability of their expression. This suggests that the strand bias structure also reflects some aspect of the chromatin structure (which in turn makes some positions advantageous for transcription): direct evidence for this is presented.

## Results and discussion

### Basic statistics about segments

The text gives results for mouse and A:T boundaries. Similar results have been obtained for human but these have not been shown. The C:G results mirror the A:T results in nearly all respects but this has not been fully explored.

**Figure 1:**
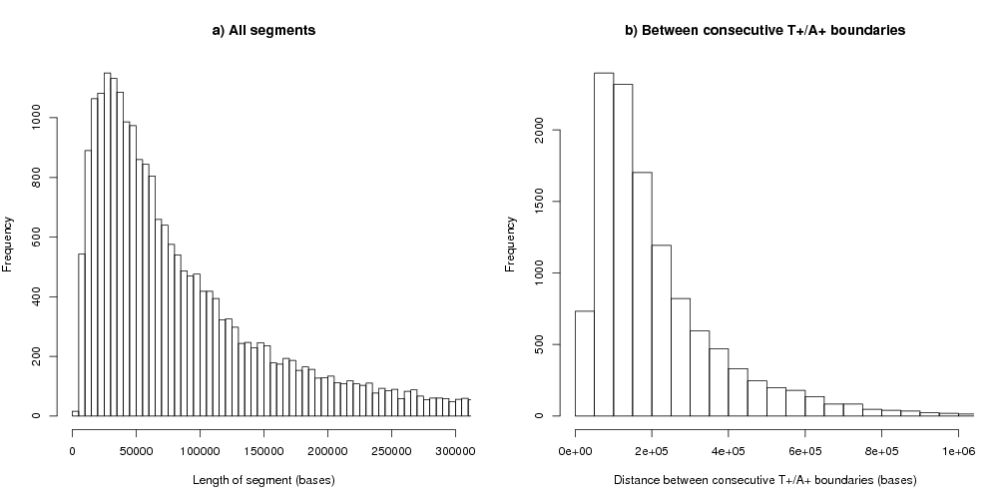
**Histogram of lengths between segment boundaries**. (a) All strand bias segments, n = 23482, median = 67289, mean = 109253. Each segment is A+ on one strand and T+ on the other. For comparison the median length of genes is 11622. (b) Distance between consecutive T+/A+ boundaries: n = 11732, median = 160700, mean = 218500, modal value around 100 k. (b) gives an estimate of the size of replicons.

The algorithm defined in the Methods section finds 23482 segments, with a median length of 67289 bases (Figure 1a) – segments bounded by either end of the chromosome are excluded from our statistics and analyses. The algorithm is not only finding boundary positions but also regions of bias between the boundaries: the median absolute AT-bias, i.e. |(A-T)/(A+C+G+T)|, of all the segments is 3.7% and the median absolute AT-skew, i.e. |(A-T)/(A+T)|, is 6.3%. The corresponding figures were 1.9% and 3.2%. for a sample of pseudo segments matched in length but with a random position in the real genome. Table 1 shows these figures with comparative results from the other control genomes analysed.

**Table 1:** Basics statistics

	Actual genome	Hybrid genome	Shuffled genome
A) Number of segments			
segments defined by algorithm	23482	8229	2013
B) Median absolute AT-bias			
segments defined by algorithm	3.68%	1.96%	0.54%
segments of random position	1.86%	0.82%	0.43%
C) Median absolute AT-skew			
segments defined by algorithm	6.26%	3.46%	0.93%
segments of random position	3.21%	1.41%	0.73%

In this table, AT-bias = median absolute (A-T)/(A+C+G+T). In this table, AT-skew = median absolute (A-T)/(A + T). Absolute values are used in this table to give meaning to the results for the random sample. The comparison between the actual and shuffled genomes confirms that the algorithm is finding real features in the real genome. The comparison between the actual and hybrid genome confirms that the average segment in the real genome is of stronger bias than a segment generated by transcription.

**Figure 2:**
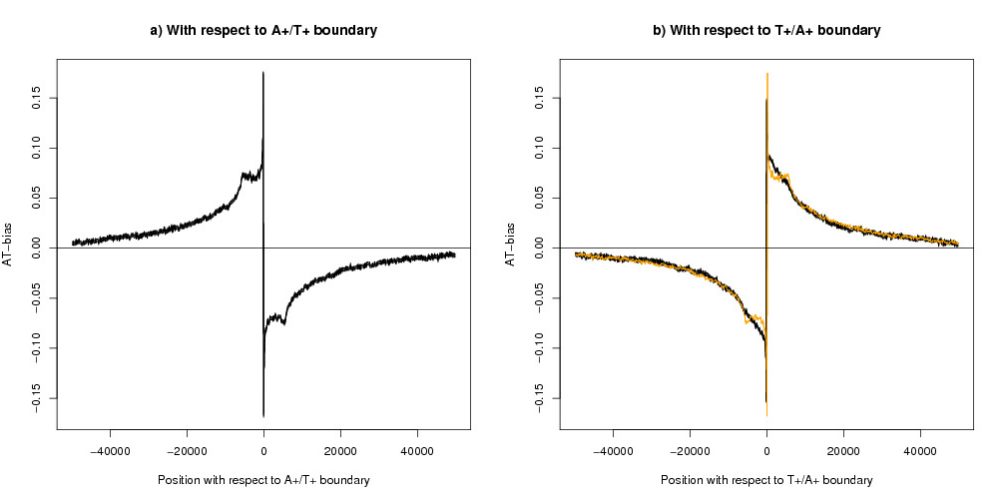
**AT-bias with respect to the segment boundaries**. Both figures show the AT-bias for 50 k bases either side of the boundary, using a moving average over 100 bases. All boundaries in the genome were used in calculating the average. The thickness of the black line shows 95% confidence limits. Comparisons with other features are shown in the following figures. a) A+/T+ boundaries (n = 11750), b) T+/A+ boundaries (n = 11753). The orange line is the mirror image of (a) and is given as a reference line. (b) does not show the shoulder feature of (a).

The A+ strand is defined to be the strand with more As than Ts, and the T+ strand is defined similarly. A DNA segment may be called the A+ segment or T+ segment, if it is clear which strand is being referred to. The average AT-bias about all the A+/T+ segment boundaries is shown in Figure 2a: the corresponding average for the T+/A+ boundary is shown in Figure 2b. AT-bias refers to the ratio (A-T)/(A+C+G+T). There is a shoulder extending to about 5000 bases in Figure 2a, which is not present in Figure 2b, and this suggests that the boundaries have different biological interpretations.

Segment lengths in the autosomal chromosomes are similar to each other with median segment lengths ranging from 62137 (chromosome 7) to 79967 (chromosome 11): the segments on the sex chromosomes are comparatively short, with median lengths 56283 for the X and 65702 for the Y. There is a relationship between AT-percentage of a segment and its length: the correlation between AT-percentage and log of the length is -0.22. Dividing segments into two according to the median AT-percentage (60%) gives a median length of 56375 for the AT-rich half and 83216 for the AT-poor half. Each segment will be A+ on one strand and T+ on the other. Because of this symmetry our later results are not a consequence of the distribution of length of segments.

### Statistical significance

**Figure 3:**
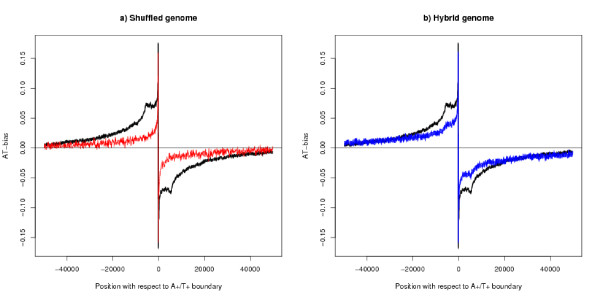
**AT-bias at the A+/T+ boundary – comparison with two control genomes**. The black line shows the actual mouse genome (11750 boundaries). a) The red line shows the shuffled genome (1017 boundaries) b) The blue line shows the hybrid genome (4124 boundaries), that is the genome has been shuffled and then the sequence for the genes has been restored at their original positions. The thickness of all lines show 95% confidence limits and all lines show moving averages over 100 bases. There is a statistically significant difference between the black and red lines and between the black and blue lines: one-tailed z-test at position 5000 bases downstream of the the boundary: a) p < 10^-50 ^n1 = 11750, n2 = 1017, z = 21. b) p < 10^-50 ^n1 = 11750, n2 = 4124, z = 20.

To assess the statistical significance of the method of finding segment boundaries, a shuffled genome was constructed by dividing the mouse genome into 100 base pieces; the pieces for each chromosome were then shuffled separately. This method has the advantage of preserving many qualities of the raw sequence including the base frequency. The same algorithm applied to the shuffled sequence finds only 2013 segments and the average bias has been plotted in Figure 3a. The analyses were repeated on two other shuffled genomes and to the scale plotted the results were identical. A statistical test also shows that the bias profiles for the real and shuffled genomes are significantly different – details in key to Figure 3a. The character of the segmentation in the real genome is different from that of the shuffled sequence: in particular the shoulder is missing from the results for the shuffled genome. Table 1 gives comparative results for the size of the bias in the segments.

### Comparison with transcription associated bias

**Figure 4:**
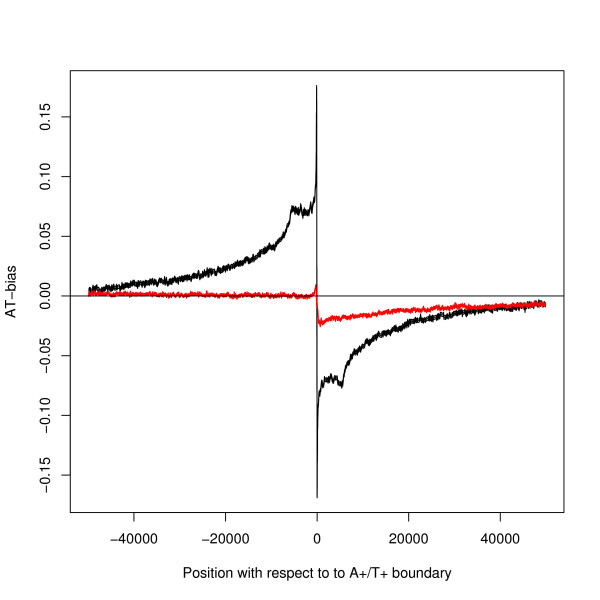
**A comparison of segment bias and transcription bias**. The black line shows the AT-bias about the A+/T+ boundary. The red line shows the AT-bias for genes aligned by their TSS: for this line the x-axis gives the position relative to the TSS. Both lines show moving averages over 100 bases. The thickness of both lines show 95% confidence limits. There is a statistically significant difference between the two lines: at position 5000 bases downstream of the boundary a two-tailed z-test gives p-value < 10^-50 ^n1 = 11750, n2 = 23941, z = -63.

The segment bias is much larger than that caused by transcription; see Figure 4 which compares the average bias at a segment boundary calculated from all 11750 A+/T+ boundaries with the average bias at a TSS (Transcription Start Site) calculated from all 23941 coding genes. A statistical test confirms that the graphs are different – see the key of Figure 4 for details. The segment bias and the transcription bias are therefore separate effects.

**Figure 5:**
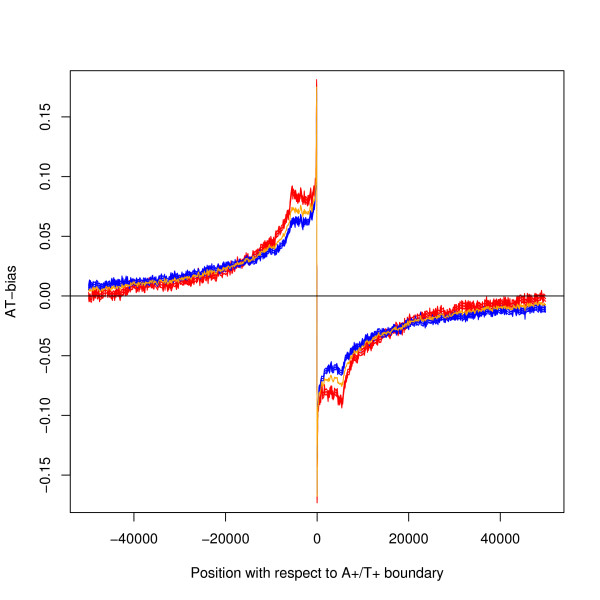
**Strand bias with respect to A+/T+ segment boundaries – comparison with no recorded transcription**. The orange line shows the AT-bias for the entire sample. The red line shows the AT-bias for the boundaries where there is no recorded transcription from coding genes for 50 kb either side of the boundary – this line is based on 4997 segment boundaries. The blue line shows the AT-bias for the boundaries where there is some recorded transcription from coding genes within the range plotted – this line is based on 6753 segment boundaries. The thickness of the red and blue lines show 95% confidence limits. All lines show moving averages over 100 bases. There is a statistically significant difference between the red and blue lines: at position 5000 bases downstream of the boundary a two-tailed z-test gives p-value < 10^-50^, n1 = 4997, n2 = 6753, z = -16.

Another direct test is to compare the average bias about A+/T+ boundaries when the boundaries are divided into those where there is no or some transcription recorded in ENSEMBL from coding genes within 50 k bases either side of the boundary. Results are shown in Figure 5, where the without-transcription graph is based on 4997 boundaries and the with-transcription graph on 6753 boundaries. The graphs are very similar, proving that the segment bias is not caused by transcription. It might be argued that the amount of transcription is under-estimated by the data used. It is common for individual genes to be found to have TSSs upstream of the recorded position. More non-coding genes are still being discovered [22] and other categories of transcription are being discovered [23]. It is possible to argue that nature is sufficiently parsimonious that transcription will be found in every part of the genome. It is therefore possible that many of the segments in the without-transcription data set contain some transcription. It is however a long stretch of the argument to say that this yet-to-be-recorded transcription will be in the right position to cause the bias shown in this graph. Figure 5 gives evidence that the data is correct: there is a slight but statistically significant difference between the with- and without-transcription graphs – see key of Figure 5 for details – implying an underlying difference in the two groups of sequences. The with-transcription graph shows a *smaller *bias near the segment boundary, which implies transcription is not the fundamental cause of this bias.

Similar analyses can be made where genes are known to be on one side of the boundary and not the other and with a given direction. These analyses all give the results that the strand bias profile on both sides of the boundary is similar to the original average shown in Figure 2a, and that when an average over all segments is made, transcription gives a only small modification of the pattern – details not shown.

Another way of analysing the bias coming from transcription is to remove the bias from everywhere except for the genes. A "hybrid genome" has been constructed by taking one of the shuffled genomes from the previous section and then copying back over this genome the actual sequences of the coding genes from TSS (Transcription Start Site) to TES (Transcription End Site) including both introns and exons in their real positions. Because of the length of introns about a third of the real genome is preserved in the hybrid genome. We then ask if the results are consistent with the hypothesis that all the strand bias in these regions comes from transcription and there is no strand bias outside these regions. The algorithm finds about a third of the segments for this genome as for the real one, 8229 as against 23482: the algorithm is searching for strand bias on a much larger scale than transcription generates. There is a difference in the profile of strand bias between the real and hybrid genomes which is proved by a statistical test – see Figure 3b and key for details.

**Figure 6:**
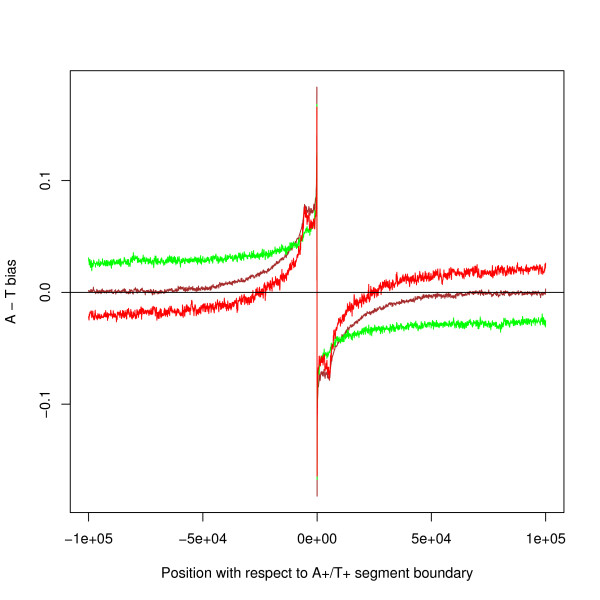
**Bias with respect to A+/T+ segment boundary – estimates for combined effect of transcription**. The brown line shows the AT-bias where there is no transcription. The green line shows the AT-bias where there is transcription in the direction from the A+/T+ boundary to the T+/A+ boundary. The red line shows the bias where there is transcription in the opposite direction. All lines show moving averages over 100 bases.

The next analysis separates the size of the bias caused by transcription from that of the segment bias. Figure 6 shows the results for all coding genes for three situations a) where there is no transcription, b) where there is transcription with the flow of the segment bias (that is in the direction from the A+/T+ boundary to the T+/A+ boundary), c) where there is transcription against the flow of the segment bias. (b) and (c) have been calculated by averaging the bias over bases in this category *at this position with respect to the segment boundary*: the calculation ignores the distance from the TSS. Bases included for the cases (b) and (c) have been excluded from (a). The data for both strands has been aggregated – the graph is therefore symmetric by definition. All three graphs show the characteristic signature at the segment boundary. The fact that the data separates into these three categories confirms that the segment bias is not caused by transcription. The segment bias dominates the transcription effect for 25 k bases either side of the segment boundary.

**Figure 7:**
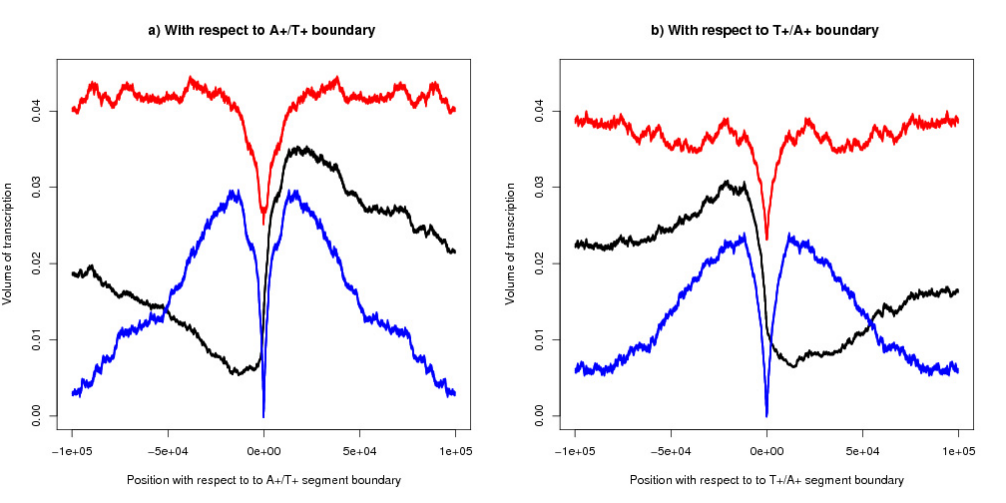
**Volume of expression by position with respect to segment boundaries – estimated from expression data**. With respect to (a) A+/T+ boundaries; (b) T+/A+ boundaries. For both graphs, the unsymmetric black line shows the volume of transcription along one strand from left to right – transcription with the flow of the segment bias is on the right for (a), and on the left for (b). The peak is about 15 kb to 20 kb downstream/upstream of the segment boundary. Corresponding data for both sides of the boundary have been averaged. The upper red line plots the sum of the amounts on the two strands and the blue line plots their absolute difference. All three lines show moving averages over 100 bases and the thickness of the lines show 95% confidence limits. For both (a) and (b), a two-tailed z-test shows that the black line at position +5000 bases is statistically different from that at position -5000: a) p < 10^-50^, n1 ~ 2058, n2 ~ 856, z = 16.5: b) p < 10^-50^, n1 ~ 2253, n2 ~ 1260, z = 16.6.

If transcription were the cause of the segment bias then the amount of transcription would be highest where the bias was highest, that is at the segment boundary. The average amount of transcription relative to the A+/T+ boundary is shown in Figure 7a. The plot uses microarray expression data to estimate the volume of expression but a similar graph is obtained from the approximation that counts one unit of transcription at each base between TSS and TES for each gene. The volume of transcription (the sum of the two strands) and its effect on strand bias (the difference between the two strands) is at a minimum in the region of the segment boundary as shown by the red and blue lines in Figure 7a – that is where the strand bias is greatest. The black line shows transcription from left to right on a single strand so that on the left hand side of the graph transcription is against the flow of the segment bias and on the right hand side it is with the flow of the segment bias. The peak flow is 15 kb to 20 kb downstream of the boundary. Although the transcription on the left hand side of the graph is less than that on the right (see the key to Figure 7 for a statistical test), it forms a non-neglible proportion of the whole. This transcription is against the flow of the bias and therefore cannot be its cause: an estimate of this proportion is given later. This Figure shows that transcription is affected by the strand bias structure. Similar arguments apply to the T+/A+ boundary (Figure 7b). These Figures imply that both A+/T+ and T+/A+ boundaries act as partial obstacles to transcription (possibly for different biological reasons).

Strand bias switch has been found within long vertebrate genes [13]: this gives direct proof that transcription is not the only cause of strand bias.

The lines of argument given above prove that the segment bias is not caused by transcription. It is therefore not a circular question to ask how transcription fits into the structure defined by the segment bias. The next series of analyses discuss this question.

### Number of genes by position in strand bias structure

**Figure 8:**
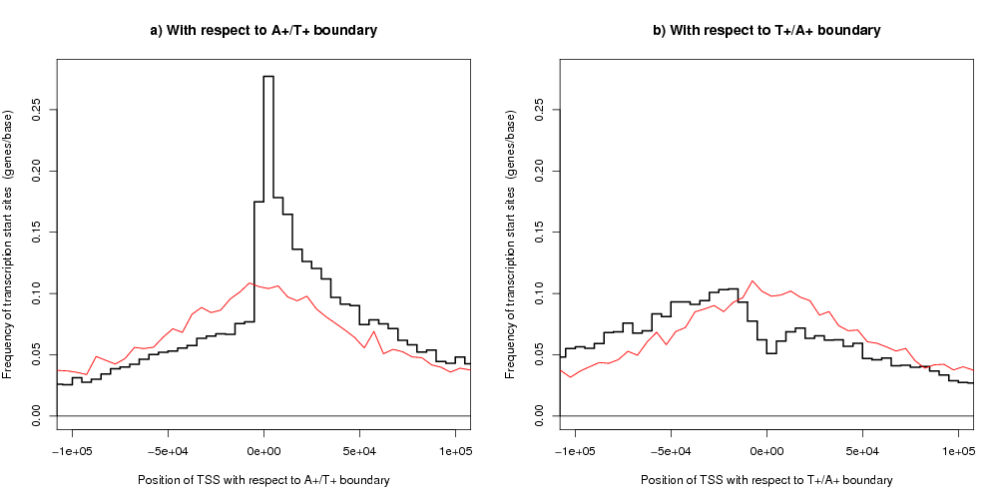
**Position of TSS with respect to the segment boundaries**. The bold black line shows results for real TSSs and the faint red line is a control plot for randomly chosen positions. (a) shows the A+/T+ boundary and (b) shows T+/A+ boundary. TSSs of genes cluster near the A+/T+ boundary and have a tendency to occur downstream of this boundary, but avoid the T+/A+ boundary. The converse applies to the TES – see Figure 9.

Transcription Start Sites cluster towards the A+/T+ boundary with a bias to the downstream side of the boundary and avoid the T+/A+ boundaries – see Figures 8a and 8b. For Figure 8a, the segment has been found which contains each TSS and the position of the TSS has been calculated with respect to the A+/T+ boundary of this segment – with upstream and downstream defined according to the strand on which the gene lies. Figure 8b shows where TSSs fall with respect to the T+/A+ boundary. This definition of the position of genes with respect to a segment boundary has also been used in the other analyses. The Figures also show a control plot where a pseudo TSS has been generated at random uniformly along the same strand of the same chromosome as the corresponding real TSS.

**Figure 9:**
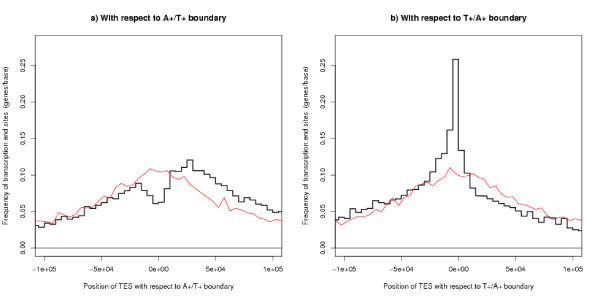
**Position of TES with respect to the segment boundaries**. The bold black line shows results for real TESs and the faint red line is a control plot for randomly chosen positions. (a) shows the A+/T+ boundary and (b) shows the T+/A+ boundary.

The opposite results apply to the Transcription End Site. The TESs are clustered towards the T+/A+ boundary with a bias to be upstream of this boundary and avoid the A+/T+ boundary – see Figures 9a and 9b. This is one place where the value of the parameter *s*, the characteristic scale in the definition of the segment finding algorithm, makes a difference. As this parameter increases the peak in the distribution of TSSs at the A+/T+ boundary remains, but the peak in the TES distribution at the T+/A+ boundary disappears, as one would expect given that the gene lengths remain fixed.

These results show a very strong bias, but few genes run from one kind of boundary to the other. The pattern is more pronounced for genes with CpG islands and for long genes (details not shown). In this context, the length of the gene is the number of bases from the TSS to the TES, that is the length of the raw unspliced mRNA.

**Figure 10:**
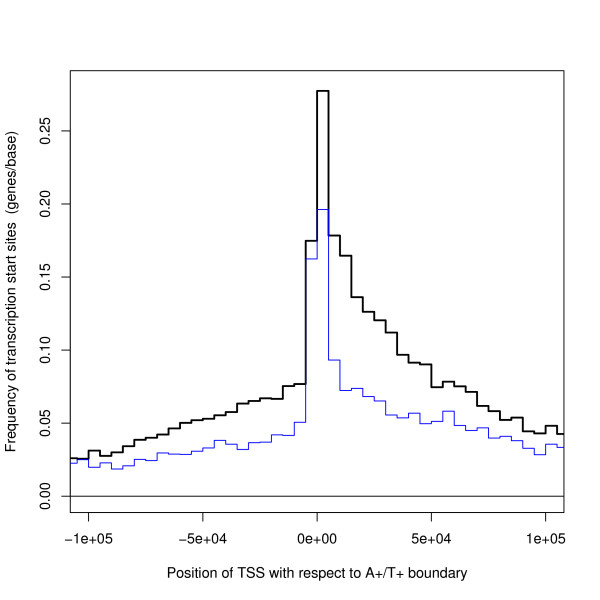
**Position of TSS with respect to the A+/T+ boundary – comparison with hybrid genome**. The black line shows results for real TSSs in the real genome and the blue line shows the results for the real TSSs in the hybrid genome. Although the lines have a common feature of a central peak, the line for the real genome is higher. The distribution of the positions of the extra TSSs found by the extra segments in the real genome is statistically significant – see Tables 2 and 3.

To discuss if Figure 8a can be explained by transcription caused strand bias, the results for the hybrid genome defined in the previous subsection are presented in Figure 10. Such an explanation appears to have some success as there is a peak in the distribution of TSSs near the segment boundary for both genomes. However, the graph for the hybrid genome is consistently lower than that for the real genome. This is partly because the hybrid genome has about one third of the segments as the real boundary. An explanation in terms of transcription does not explain the cause of all the other segment boundaries of the real genome and why the extra segment boundaries are finding TSSs in a similar position with respect to the boundary as in the hybrid genome. The analysis for the real genome finds 5908 more TSSs than for the hybrid genome (in the range plotted) which are split 1947 upstream and 3961 downstream (Table 2). The number of these extra TSSs in the region 50 kb downstream of the A+/T+ boundary is more than expected for random positions (Table 3). Both results are statistically highly significant – see Tables 2 and 3.

**Table 2:** Number of genes – comparisons with hybrid genome – upstream versus downstream

	Genome	Number of TSSs upstream	Number of TSSs downstream	Total	Proportion
	i	ii	iii	iv = ii + iii	v = iii/iv

A	real	5738	10033	15771	63.6%
B	hybrid	3791	6072	9863	61.6%
C	A – B	1947	3961	5908	67.0% (X)

Columns ii and iii refer to the number of TSSs within 100 kb of the A+/T+ boundary, upstream or downstream respectively. The figure of 67.0% (X) is significantly different from 50.0% using a binomial distribution approximated by the normal distribution, p < 10^-50^, n = 5908, z = 26, one tailed test.

**Table 3:** Number of genes – comparisons with hybrid genome – third quarter comparison

	Genome	Number of TSSs Quarters 1,2 4	Number of TSSs Quarter 3	Total	Proportion
	i	ii	iii	iv = ii + iii	v = iii/iv

A	real	8803	6968	15771	44.2%
B	hybrid	5939	3924	9863	39.8%
C	A – B	2864	3044	5908	51.5% (X)
D	real (random)	9362	4386	13748	31.9% (Y)

Quarter 3 is the region up to 50 kb downstream of the A+/T+ boundary. Quarters 1,2, 4 are the other parts of the region between 100 kb upstream and 100 kb downstream of this boundary. Row D refers to the control analysis in which TSSs are replaced by an equal number of random positions (the red line of Figure 9a). The figure of 51.5% (X) is significantly different from the figure 31.9% (Y) using a binomial distribution approximated by the normal distribution and a t-type test, p < 10^-50^, n1 = 5908, n2 = 13478, z = 25, one tailed test.

### Length of genes by position in strand bias structure

**Figure 11:**
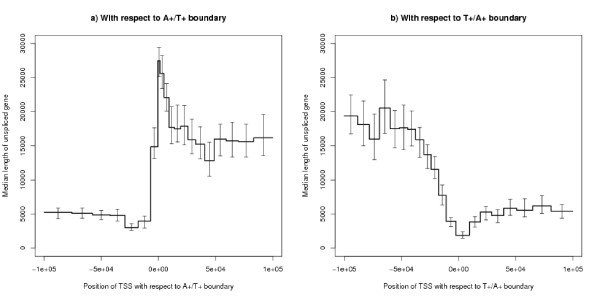
**Median length of gene by position of TSS with respect to the segment boundaries**. The bins have been defined by the quantiles of the distribution within the range plotted. The error bars show 95% confidence ranges using Hettmansperger-Sheather's method. (a) shows the A+/T+ boundary and (b) shows the T+/A+ boundary. a) in each bin, *n *~ 788; b) in each bin, *n *~ 655.

Genes starting near the A+/T+ boundary tend to be long and those starting on the T+ segment are much longer than on the A+ segment (Figure 11a). Conversely, genes starting near the T+/A+ boundary tend to be very short (Figure 11b). The ratio between the peak of Figure 11a and the minimum of Figure 11b is larger than five.

### Gene expression by length of gene

**Figure 12:**
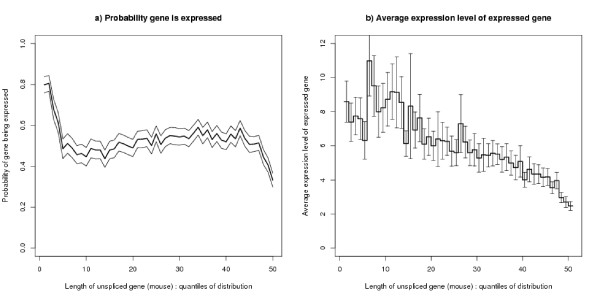
**Gene expression by length of gene**. The bins for the x-axis are the quantiles of the length distribution for those genes which have expression data. There are 50 bins each containing 2% of the distribution: in each bin *n *~ 277. The 10%, 50% and 90% quantiles are gene lengths 1211, 14975 and 91253. 95% confidence limits are shown by the upper and lower lines in (a) and the error bars in (b). a) Probability that a gene is expressed by length of gene: The bulk of the figure shows that on average increasing length of gene implies an increasing probability that the gene is expressed. However, extremely short genes have a high probability of always being expressed and very long genes are less likely to be expressed. b) Average expression level of genes that are expressed by length of gene: The expression level is quoted in arbitrary linear units.

The relationship between the probability that a gene is expressed and the length of the gene is shown in Figure 12a. The x-axis of the graph shows quantiles of the length distribution. Very short genes are nearly always expressed. For the bulk of the graph the probability of expression slowly increases with gene length. The very longest genes have a low probability of expression. Figure 12b shows the average expression level of expressed genes by length of gene (using variable b defined in the Methods section). Generally expression levels decrease with length of the gene. Both figures show more structure than previously reported results [24-28].

### Gene expression by position in strand bias structure

**Figure 13:**
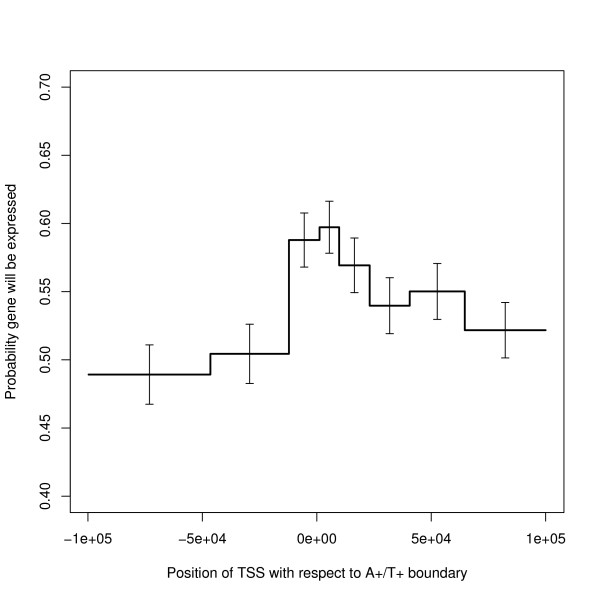
**Probability of a gene being expressed by position of TSS with respect to the A+/T+ boundary**. The bins have been defined by the quantiles of the distribution within the range plotted: in each bin, n ~ 1152. The error bars show 95% confidence limits. This shows a strong peak near the segment boundary and a long range asymmetry about the boundary.

**Figure 14:**
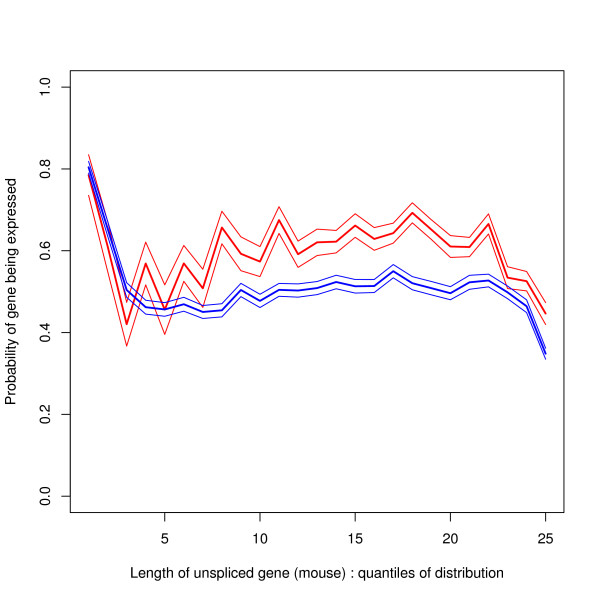
**Probability of a gene being expressed by length of gene – split by position of TSS with respect to A+/T+ boundary**. The upper red line refers to genes whose TSS is within 5 k bases upstream of the A+/T+ boundary and 15 k bases downstream of this boundary and the lower blue line refers to genes whose TSS falls outside this range. The analysis is based on 2532 genes (red line) and 11291 genes (blue line). This figure explains why genes with TSS near this boundary are often expressed, despite the fact that these genes tend to be long genes (Figure 11a) and long genes tend to be less often expressed (Figure 12a). The plot shows plus and minus one standard error.

Given these results it is to be expected that the probability that a gene is expressed (and its expression level if expressed) varies with the position of the TSS and TES within the strand bias structure. This is borne out by direct analysis. However, Figure 13 shows that the probability that a gene is switched on when the TSS is near the A+/T+ boundary is *not *what would be expected from combining the effects of Figures 11a and 12a. The explanation is that, for most gene lengths, the positional effect is stronger than the length effect. To clarify the point, Figure 14 shows the probability of expression by length (as in Figure 12a) split by the position of the TSS with respect to the A+/T+ boundary, separating out genes starting between 5 k bases upstream and 15 k bases downstream of this boundary. Figure 13 also shows that genes starting in the segment downstream of the boundary (i.e. the T+ segment) are more likely to be expressed than those starting in the upstream segment (i.e. the A+ segment). It is difficult to explain this graph by transcription caused bias: the right hand side of this figure shows that the strand bias segment starts further upstream of the TSS for those genes which are less often expressed.

The expression level of a gene (if it is expressed) shows a much weaker relationship with position of the TSS (or TES) with respect to the segment boundary. Because of the larger statistical uncertainties, we have reported a comparison between a) the genes which are within a T+ segment (with the flow) and b) those genes within an A+ segment (against the flow). In both cases genes which cross either an A+/T+ boundary or a T+/A+ boundary have been omitted. Most of these excluded genes are extremely long. Results are given in Table 4. The average level of expression of a gene that is expressed for group a) is less than that for group b), which is opposite to what one would expect.

**Table 4:** Expression levels on different segments

segment	sample size	probability expressed	level of expressed gene	average expression	median length
1	2	3	4	5	6

T+:	7784	0.56	6.03	3.75	17606
A+:	4080	0.50	7.10	3.62	6145

Gene crossing either A+/T+ or T+/A+ boundary have been omitted from the sample. Genes on a T+ segment are with the flow of the bias and those on an A+ segment are against the flow. For an individual gene the variable in column 5 is the product of the variables in columns 3 and 4. Columns 4 and 5 are in arbitrary linear units. Genes with the flow of the bias have a higher probability of being expressed (column 3) than those against the flow, but their level of expression when expressed is smaller (column 4). Genes against the flow are much shorter than those with the flow (column 6). The differences between the segment types are statistically significant for columns 3 and 4 using a two-tailed t-test, p values ~ 2.10^-16 ^and 9.10^-7 ^respectively. The difference between the segment types in column 5 is not statistically significant.

### Proportion of transcription with the flow of the strand bias

The proportion of DNA that is transcribed "with the flow" of the strand bias has been calculated as follows. As a gene may cross several segment boundaries, the number of bases on the T+ strand and the number on the A+ strand were counted for each gene. The number of bases was then totalled by strand. The result is that the number of transcribed bases on the T+ strand is 77% of all transcribed bases. If the number of bases is weighted by the average expression level of the gene then the proportion rises to 82%. If transcription was the cause of the bias one would expect a value close to 100%.

### Discussion of three previous papers

Touchon *et al*. [9] is one of a number of papers (compare [8]) to report an average strand bias when sequences are aligned by the transcription start site or end site: for example their AT-skew measure, (A-T)/(A+T), jumps to about 5% at the TSS. The main argument that these are transcription caused biases was the comparison with the near absence of average bias in the upstream region. When allowance is made for the different measures of the bias the result is similar to Figure 4. However, the same figure shows that the strand bias discussed here is different in kind from the transcription associated strand bias.

**Figure 15:**
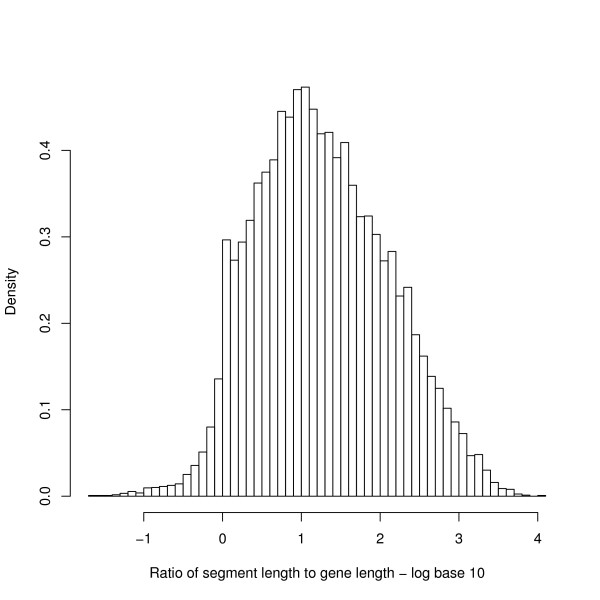
**Histogram of ratio of length of containing segment to gene length**. The median ratio is 16 and there is an apparent boundary to the distribution at a ratio around 1. The interquartile range of the ratio is [4.4, 72]. The x-axis is plotted using logs to base 10. The number of genes in the plot is 23878. The segment bias operates on a larger scale than the transcription bias.

Green *et al*. [14] analysed the bias for individual genes and found that the "maximal segments" around the genes defined by the strand bias roughly approximate to the transcribed unit. We have therefore compared the length of the strand bias segments under our definition with the length of the gene. Genes have a median length of 11622 (n = 23941) and the segments analysed in this paper have a median length of 67289 (n = 23482). An individual gene can cross several segment boundaries, but if a gene is defined to be "in" the segment which contains the base midway between the ends, then the median length of the containing segment is 180900 (for 23878 matched genes). The median ratio of the containing-segment length to gene length is 16. Figure 15 shows a histogram of this ratio on a log scale. There is an apparent boundary in the figure where the ratio is near one (i.e. log(value) = 0), and we expect that this has biological significance. However, the segments of this paper are normally much larger than the transcription units they contain.

**Figure 16:**
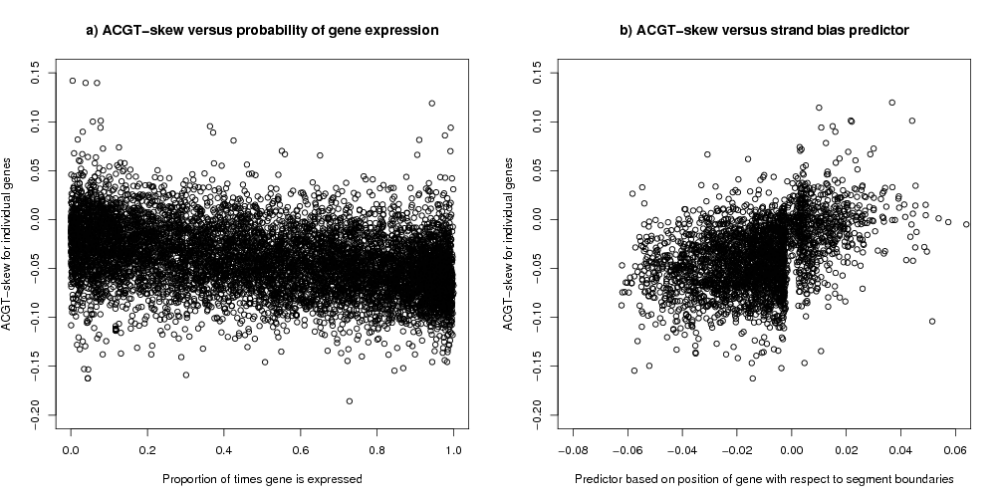
**ACGT-skew for individual genes – long genes**. The y-axis is the ratio ((A+C)-(T+G))/(A+C+G+T)) for introns (and also excluding the 50 bases at each end of the intron) as used in [16]. The plot is restricted to genes of at least 10 k bases. a) ACGT-skew for individual genes by proportion of times gene is expressed: The plot shows a correlation of -0.39 (n = 8352). Even for seldom expressed genes there is an average negative bias. b) ACGT-skew for individual genes by segment bias predictor: The plot excludes genes which extend more than 100 k bases from both A+/T+ and T+/A+ boundaries, and this explains the gap in the plot. The correlation is 0.43 (n = 9040). The predictor does not use any information about transcription other than the position of the gene with respect to the segment boundaries. In particular, no information about transcription bias is used in calculating the predictor.

If transcription causes a strand bias, it would be expected that this effect would be roughly proportional to expression level and Majewski [16] noted such a relationship: he defined a variable ("ACGT-skew") as the ratio ((*A *+ *C*) - (*T *+ *G*))/(*A *+ *C *+ *G *+ *T*)) for introns (and excluding the 50 bases at each end of the intron). He found a correlation of 0.28 between log of the expression level and this ACGT-skew in a sample of 374 house keeping genes – the sample being chosen to represent genes transcribed in the germ line. We have obtained comparable results from similar analyses using the same definition of ACGT-skew, by being similarly selective in the choice of genes – restricting the number of genes by the probability of being expressed greater than *x*, (where *x *~ 0.99). As any biological cause of strand bias will be a statistical process, the average bias per base will be more predictable for the longer genes. For this reason, results are more consistent for longer genes (those longer than 10 k bases). When all genes are included in the analysis, the correlation degrades but only slightly. The correlation between the ACGT-skew and log of the amount of gene expression for all genes in the dataset is -0.19 (n = 12255) and for long genes -0.27 (n = 8352). The measure for gene expression is an average over all experiments and therefore tissue types. A stronger correlation of -0.28 (n = 12255) is obtained between the ACGT-skew and the proportion of times a gene is expressed. For long genes the correlation is -0.39 (n = 8352): this is plotted in Figure 16a. It is possible to say that these analyses have used poor proxies for expression in the germ line and therefore have obtained poor correlations with strand bias, and this may be part of the explanation of the results. However, in our interpretation much of the relationship comes from the tendency of highly expressed genes to be near the segment boundary. In Figure 16a there is an average strand bias for all categories of genes including those that are seldom expressed (i.e. tissue specific) and it is unreasonable to suppose that these seldom expressed genes are expressed in the germ line.

In many cases one can get a better predictor of the strand bias of individual genes, merely by using the knowledge of the position of the gene with respect to the segment boundaries defined here. For each base take the nearest A+/T+ or T+/A+ boundary and associate with this base the average AT-bias for that position using the line from Figure 2a or 2b. The predictor is the average of these scores over the length of the gene. Far away from the boundary, this predictor is not useful and the analysis excludes those genes (about a quarter of the whole) which extend beyond 100 k bases from either type of boundary. The correlation between the ACGT-skew and this predictor is 0.24 (n = 14195). If one considers only genes longer than 10 kb the correlation is 0.43 on 9040 genes – see Figure 16b – which is a better correlation than Majewski's result on 374 genes. If the bias is measured by the average AT-bias over the whole length of the gene from TSS to TES, the correlation with the predictor is 0.31 (n = 18479) and 0.48 (n = 9040) for long genes. A predictor based on the red and green lines of Figure 6 and its T+/A+ equivalent performs slightly better than the one described.

### Strand bias and DNA replication

The direct cause of the strand bias observed in this paper is not known but an appealing theory is that the strand bias comes from the mechanism of DNA replication and the A+/T+ boundaries are origins of replication. There are several reasons to think this may be so:- strand asymmetries of this type have been observed at origins of replication in bacterial and viral genomes i.e. the leading strand is *G*+ and often *T*+ [3-6], and as these references explain there is an asymmetry between the strands in the mutation/repair processes which gives a physical explanation of the strand bias. This process can be expected to affect almost all of the genome. Touchon *et al *[11] examined the region 100 kb either side of a number of human origins of replication and found this effect in six out of nine examples. Although this statistic is inconclusive, the same research goup has developed the argument in [19,29] and the theory remains attractive.

The finding that 82% of transcription is with the flow of the strand bias adds weight to this suggestion. In almost all prokaryotes studied there is a bias in that the direction of transcription is the same as that of replication [30]. A possible reason for this is to avoid a molecular collision between the replication and transcription machinery. A simple gene count does not suggest a very strong bias, e.g. 55%:45% for *E. coli *[31], but the bias is stronger when the volume of expression is taken into account. However, influences such as essentiality [32] or transcription interruption [33] are involved for *E. Coli *as well as expression levels so that less than 100% of gene expression "with the flow" is plausible when considering the relationship between replication and transcription for mouse. Experimental work [34] has shown that transcription against the flow of the replication machinery is associated with replication fork pause and with chromosome recombination which would be generally detrimental to the organism. I am grateful to Sascha Ott for this line of argument (personal communication, 2006).

Estimates for the size of replicons (the region of DNA controlled by one origin of replication) fall into two groups: those agreeing with the traditional view that replicons are comparatively small: around 50 kb to 300 kb [35], around 100 kb for animals [36], common sizes between 50 kb and 100 kb [37]: and those quoting a larger size: mammalian average up to 500 kb [38], 1 Mb–2 Mb [11,29], mean around 1.2 Mb [19]. In our analyses, a replicon extends from one T+/A+ boundary to the next and has a median size 160 kb and a mean size 220 kb. The modal value is around 100 kb (Figure 1b). Our analyses use the unknown parameter *s *= 50*k*: this is a plausible value (because the results are consistent and there is a symmetry in the positions of TSS and TES of genes, Figures 8 and 9). The value of *s *is uncertain, but these results will be upper bounds and they argue against the larger estimates in the literature.

Another model for the relationship with DNA replication is that the direct cause of strand bias is transcription, but the placement of genes and direction of transcription is controlled by the need to keep transcription and replication in the same direction. This model has been proposed [39] for prokaryotes (which have only one or very few origins of replication) and where the genome is much more compact, transcription is less associated with a single gene, and all processes are in the germ-line. In the present context, this model is ruled out by the difference between the bias at segment boundaries and TSSs (Figure 4).

### Strand bias and chromatin organisation

**Figure 17:**
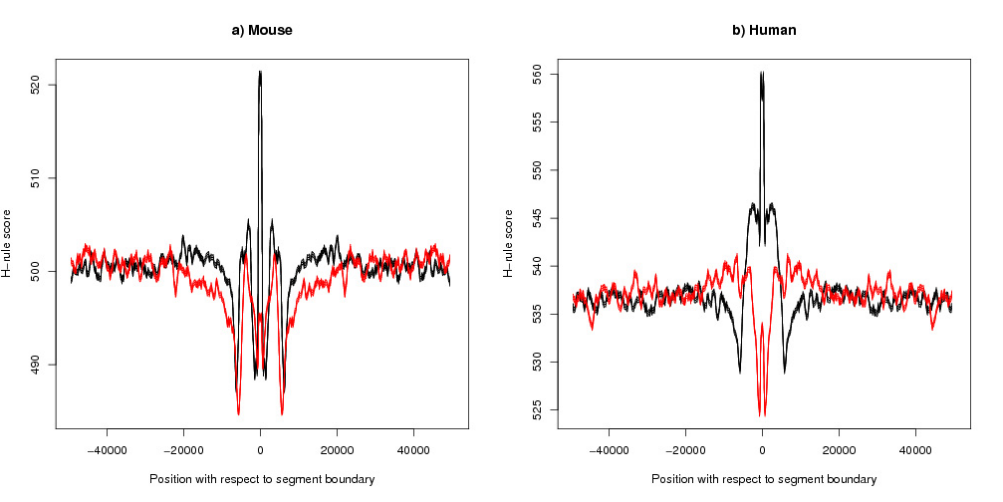
**H-rule measure by position with respect to segment boundary**. The black line (line with peak) gives the value of the H-rule measure with respect to the A+/T+ boundary, and the red line with respect to the T+/A+ boundary. The data from both sides of the boundary have been averaged. The thickness of both lines shows 95% confidence limits. The black line has a peak at the boundary but the red line does not. This suggests that the A+/T+ boundary is a region which binds to the nuclear proteins of the matrix, in particular, SATB1. a) Mouse: b) Human (genome assembly NCBI35). For both plots the difference between the lines at the boundary is statistically significant, two-tailed z-test: a) p < 10^-50^, n1 = 11750, n2 = 11753, z ~ 104: b) p < 10^-50^, n1 = 12375, n2 = 12369, z ~ 96.

An explanation in terms of DNA replication does not explain the various relationships that have been observed between the strand bias and the placement of genes, their length, the chance that a gene is switched on and the expression level of genes. All this calls for a unifying explanation, which we suggest is to be found in the physical structure of the chromatin. Similar results (although for much larger domains) lead Huvet *et al*. [19] to say "[these results] ... present a high level of organization, possibly mediated by the chromatin structure." It is likely that the physical position of the gene in the chromatin would affect the ease of transcription and would be used to control transcription. The reasons that make a position an origin of replication may also play a special role in controlling transcription. This description would fit with the three dimensional descriptions of DNA which include loop attachments [40,41]. It has been suggested that matrix attachment regions are at origins of replication [42,43]. To test the hypothesis that there are matrix attachment regions at the A+/T+ boundaries, we have used a version of the H-rule, which looks for regions containing long runs of Hs (that is A, C or T). The nuclear protein SATB1 is known to bind to this kind of sequence [44,45]. The measure used is the number of occurrences of a motif of 20 consecutive Hs in a window of 1000 bases on both strands allowing two mismatches and allowing overlaps between motifs. There is a sharp narrow peak for this measure at the A+/T+ boundary (Figure 17a) and the valleys surrounding the peak support this interpretation. This is not an artefact of the underlying definitions, because there is no such peak at the T+/A+ boundary (Figure 17a) and a control plot based on the shuffled genome shows no structure at either boundary – details not shown. We cannot explain the valley structure around the T+/A+ boundary, but it suggests some biological feature. The corresponding plots for human, Figure 17b, have a more prominent pattern at both A+/T+ and T+/A+ boundaries. Although the S/MAR prediction rules are not reliable for an individual sequence, the signal given by the average is indicative of a biological feature. For comparison, this measure averages around 480 for DNA randomly chosen from mouse and a sample of known S/MARs showed a peak around 540 – see [46] for a discussion.

## Conclusions

We have shown the mouse genome has a strand bias structure consisting of segments of alternating bias. These segments are much larger than coding genes. These segments influence the placement of genes, their length, the probability that a gene is expressed, and the size of the expression level. These effects are not caused by transcription even though transcription itself causes a strand bias effect. Although the direct cause of the bias may be DNA replication, the strand bias in question represents a further biological structure, such as the spatial organisation of the chromatin. The H-rule analysis gives direct evidence for this proposal.

## Methods

### Definition of strand bias segments

A region may be mostly T+ but contain an A+ sub-region. This region might be defined to be one T+ segment or one A+ segment and two T+ segments. In order to choose between these possibilities, we use a parameter, *s *bases, called the characteristic scale, to show the size of the feature of interest. At an A+/T+ segment boundary, there should be more *A*s than *T*s in the window upstream of the boundary and more *T*s than *A*s in the window downstream of the boundary. The simplest operational definition would be the position where the sum of these counts is a local maximum, but this definition would depend on the exact distribution of bases around the far edge of the window as much as at the near edge. Exponentially moving averages have been used to soften the effect of the window boundary at the far edge from the candidate segment boundary. To prevent the size of the bias being an artefact of the AT% of the region, the average bias is defined as the weighted bias divided by the weighted count of the number of A and T bases. The absolute value average bias is required to be greater than a threshold value in both upstream and downstream windows, thus allowing an element of statistical significance to be included. The condition that T+/A+ and A+/T+ boundaries alternate has been imposed by removing all but the most extreme of consecutive boundaries of the same type. We have experimented with other ways of selecting the boundaries and obtained results similar in kind, but the adopted procedure has the advantage of not imposing a hard limit on the segment size.

The following equations give a precise description of the method. The exponential weighting factor *w *is defined by 1 - *w *= 2/(*s *+ 1). With this value of *w*, a window size *s *contributes 85% of the sum of the weights in an infinite window. However, to minimise a small artefact coming from the finite size of the window, a larger sized window, *N*, has been chosen as *N *= 2*s*. This means that any segment boundary must be at least *N *bases from each end of the chromosome. For each base *i *in a chromosome, where *N *≤ *i *≤ *G *- *N *and *G *is the chromosome length, we calculate a window score, *S*_*L*_[*i*], for the window extending *N *bases to the left, and likewise for the window extending *N *bases to the right *S*_*R*_[*i *+ 1]. This window score is defined by the following steps:

Let *j *be any position in the chromosome, then define *m*[*j*] and *c*[*j*] as:

*m*[*j*] = 1, if the base at position *j *is A; *m*[*j*] = -1, if the base is T; and *m*[*j*] = 0 for other possibilities; and 

*c*[*j*] = 1, if the base at position *j *is A or T; and *c*[*j*] = 0 for other possibilities.

Variables will be defined in pairs with suffix *L *refering to the left hand window and suffix *R *to the right hand window. The weighted bias in each window is defined to be:

$$\begin{array}{ccc} B_{L}[i]=\sum_{k=0}^{N-1}m[i-k]w^{k} & \text{and} & B_{R}[i+1]=\sum_{k=0}^{N-1}m[i+1+k]w^{k} \end{array}$$

and the weighted count of the number of A and T bases is defined to be:

$$\begin{array}{ccc} C_{L}[i]=\sum_{k=0}^{N-1}c[i-k]w^{k} & \text{and} & C_{R}[i+1]=\sum_{k=0}^{N-1}c[i+1+k]w^{k} \end{array}$$

The window score in each window is then defined as the average bias:

*S*_*L*_[*i*] = *B*_*L*_[*i*]/*C*_*L*_[*i*]          and          *S*_*R*_[*i *+ 1] = *B*_*R*_[*i *+ 1]/*C*_*R*_[*i *+ 1]

A threshold for each window is defined by:

$$\begin{array}{ccc} Z_{L}[i]=r/\sqrt{C_{L}[i]} & \text{and} & Z_{R}[i+1]=r/\sqrt{C_{R}[i+1]} \end{array}$$

where *r *= 2. The value of r gives a measure of statistical control.

Candidate A+/T+ boundaries are then chosen as those positions *i *where

*S*_*L*_[*i*] > *Z*_*L*_[*i*]         and         S_R_[i + 1] < -Z_R_[i + 1]

and candidate T+/A+ boundaries as those positions *i *where

*S*_*L*_[*i*] < -*Z*_*L*_[*i*]          and          S_R_[i + 1] > Z_R_[i + 1]

For these positions we define a measure:

*D*[*i*] = *S*_*L*_[*i*] - *S*_*R*_[*i *+ 1]

As a convenience in the computations, if any candidate positions of the same type are within 100 bases of each other we immediately chose the one with the more extreme value of D[i]. The A+/T+ and T+/A+ candidate positions are then ordered by position. For each group of consecutive A+/T+ boundaries the one with the greatest (most positive) value of D[i] is selected and for each group of consecutive T+/A+ boundaries the one with the least (most negative) value of D[i] is chosen. The resulting boundary positions define the strand bias segments.

We are interested in large scale effects. The following values of the parameters have been used for the results presented in this paper: *s *= 50*k *bases, *w *= 2/(*s *+ 1) and window size *N *= 100*k *bases. A wide range of values have been analysed and have been found to give similar results. As the scale is increased, the algorithm picks out fewer but more extreme examples of segments which are longer and show greater bias.

### Data sources

Although the expression level of a gene is affected by a large number of variables (age of the organism, the position within the organism, phase of the cell cycle, environmental stress, etc.) and is highly variable, it is useful to consider average expression levels. Three variables have been used: a) the probability of expression, (number of experiments in which a gene is expressed divided by number of experiments), b) the average expression level if it is expressed (sum of the gene's expression levels over all experiments divided by number of experiments in which it is expressed), and c) its average expression level (sum of gene's expression levels divided by total number of experiments): for an individual gene *a *× *b *= *c*. These have been estimated from the data deposited with GEO [47]: a microarray chip was chosen (i.e. a GEO platform with a GPL number) and every corresponding GSM file (i.e. set of results) was used which had rows for all probes and columns for probe-id, expression-level and either present-absent-call or detection-probability. The column present-absent-call was used if available, otherwise the detection-probability was converted to a call using a threshold of 0.04. The expression level for each chip has been recalibrated by setting the expression level for absent probes to zero, and normalising the total expression level of the present probes on the chip to unity. The platform was chosen to be an Affymetrix chip and the probes have been associated with an ENSEMBL gene using the match with the probe-id given by ENSEMBL [48]. Where several probes have been matched to a gene, the average value for the probes has been used: where one probe has been matched to several genes, the call for the probe has been given to each gene but the expression level for the probe has been shared amongst the genes.

The data for the chromosomal sequence, the list of genes and their TSSs and TESs has been taken from ENSEMBL, which means that for each gene the transcribed unit has been taken to be the union of all alternative transcripts. The analysis includes all protein coding genes but excludes mitochondrial genes.

The mouse analysis is based on sequence assembly NCBIM36 and GEO platform GPL339, where 1744 GSM files had sufficient data to be used. This platform has 22690 probe-sets. Information on mouse genes was taken from ENSEMBL 45.

## Abbreviations

AT-bias = (A-T)/(A+C+G+T); AT-skew = (A-T)/(A+T); ACGT-skew = ((A-T)+(C-G))/(A+C+G+T). TSS = Transcription Start Site; TES = Transcription End Site. The A+ strand is the strand with more As than Ts, and T+ strand is defined similarly. A DNA segment may be called the A+ segment or T+ segment, if it is clear which strand is being referred to.

## References

[B1] Rudner R, Karkas JD, Chargaff E (1968). Separation of B. subtilis DNA into complementary strands. III. Direct Analysis. Proc Natl Acad Sci USA.

[B2] Karlin S, Campbell AM, Mrazek J (1998). Comparative DNA analysis across diverse genomes. Annu Rev Genet.

[B3] Lobry JR (1996). Asymmetric substitution patterns in two DNA strands of bacteria. Mol Biol Evol.

[B4] Grigoriev A (1998). Analyzing genomes with cumulative skew diagrams. Nucleic Acids Res.

[B5] Mrazek J, Karlin S (1998). Strand compositional asymmetry in bacterial and large viral genomes. Proc Natl Acad Sci USA.

[B6] Tillier ER, Collins RA (2000). The contributions of replication orientation, gene direction, and signal sequences to base-composition asymmetries in bacterial genomes. J Mol Evol.

[B7] Fujimori S, Washio T, Tomita M (2005). GC-compositional strand bias around transcription start sites in plants and fungi. BMC Genomics.

[B8] Aerts S, Thijs G, Dabrowski M, Moreau Y, De Moor B (2004). Comprehensive analysis of the base composition around the transcription start site in Metazoa. BMC Genomics.

[B9] Touchon M, Nicolay S, Arneodo A, d'Aubenton-Carafa Y, Thermes C (2003). Transcription-coupled TA and GC strand asymmetries in the human genome. FEBS Lett.

[B10] Touchon M, Arneodo A, d'Aubenton-Carafa Y, Thermes C (2004). Transcription-coupled and splicing-coupled strand asymmetries in eukaryotic genomes. Nucleic Acids Res.

[B11] Touchon M, Nicolay S, Audit B, Brodie of Brodie EB, d'Aubenton-Carafa Y, Arneodo A, Thermes C (2005). Replication-associated strand asymmetries in mammalian genomes: towards detection of replication origins. Proc Natl Acad Sci USA.

[B12] Niu DK, Lin K, Zhang DY (2003). Strand compositional asymmetries of nuclear DNA in eukaryotes. J Mol Evol.

[B13] Wang HF, Hou WR, Niu DK (2007). Strand compositional asymmetries in vertebrate large genes. Mol Biol Rep.

[B14] Green P, Ewing B, Miller W, Thomas PJ, Green ED, NISC Comparative Sequencing Program (2003). Transcription-associated mutational asymmetry in mammalian evolution. Nat Genet.

[B15] Qu HQ, Lawrence SG, Guo F, Majewski J, Polychronakos C (2006). Strand bias in complementary single-nucleotide polymorphisms of transcribed human sequences: evidence for functional effects of synonymous polymorphisms. BMC Genomics.

[B16] Majewski J (2003). Dependence of mutational asymmetry on gene-expression levels in the human genome. Am J Hum Genet.

[B17] Louie E, Ott J, Majewski J (2003). Nucleotide frequency variation across human genes. Genome Res.

[B18] Glusman G, Qin S, El-Gewely MR, Siegel AF, Roach JC, Hood L, Smit AFA (2006). A third approach to gene prediction suggests thousands of additional human transcribed regions. PLOS Comput Biol.

[B19] Huvet M, Nicolay S, Touchon M, Audit B, d'Aubeton-Carafa Y, Arneodo A, Thermes C (2007). Human gene organization driven by the coordination of replication and transcription. Genome Res.

[B20] Evans KJ (2008). Genomic DNA from animals shows contrasting strand bias in large and small subsequences. BMC Genomics.

[B21] Messer PW, Arndt PF (2006). CorGen – measuring and generating long-range correlations for DNA sequence analysis. Nucleic Acids Res.

[B22] Griffiths-Jones S, Moxon S, Marshall M, Khanna A, Eddy SR, Bateman A (2005). Rfam: annotating non-coding RNAs in complete genomes. Nucleic Acids Res.

[B23] Werner A, Schmutzler G, Carlile M, Miles CG, Peters H (2007). Expression profiling of antisense transcripts on DNA arrays. Physiol Genomics.

[B24] Castillo-Davis CI, Mekhedov SL, Hartl DL, Koonin EV, Kondrashov FA (2002). Selection for short introns in highly expressed genes. Nat Genet.

[B25] Eisenberg E, Levanon EY (2003). Human housekeeping genes are compact. Trends Genet.

[B26] Chiaromonte F, Miller W, Bouhassira EE (2003). Gene length and proximity to neighbors affect genome-wide expression levels. Genome Res.

[B27] Stenoien HK, Stephan W (2005). Global mRNA stability is not associated with levels of gene expression in Drosophila melanogaster but shows a negative correlation with codon bias. J Mol Evol.

[B28] Li SW, Feng L, Niu DK (2007). Selection for the miniaturization of highly expressed genes. Biochem Biophys Res Commun.

[B29] Brodie Of Brodie EB, Nicolay S, Touchon M, Audit B, d'Aubenton-Carafa Y, Thermes C, Arneodo A (2005). From DNA sequence analysis to modelling replication in the human genome. Phys Rev Lett.

[B30] Mclean MJ, Wolfe KH, Devine KM (1998). Base composition skews, replication orientation, and gene orientation in 12 prokaryote genomes. J Mol Evol.

[B31] Blattner FR, Plunkett G, Bloch CA, Perna NT, Burland V, Riley M, Collado-Vides J, Glasner JD, Rode CK, Mayhew GF (1997). The complete genome sequence of Escherichia coli K-12. Science.

[B32] Rocha EP, Danchin A (2003). Essentiality, not expressiveness, drives gene-strand bias in bacteria. Nat Genet.

[B33] Price MN, Alm EJ, Arkin AP (2005). Interruptions in gene expression drive highly expressed operons to the leading strand of DNA replication. Nucleic Acids Res.

[B34] Prado F, Aguilera A (2005). Impairment of replication fork progression mediates RNA polII transcription-associated recombination. EMBO J.

[B35] Huberman JA, Rigss AD (1968). On the mechanism of DNA replication in mammalian chromosomes. J Mol Biol.

[B36] Lewin B (2000). Genes VII.

[B37] Hou WR, Wang HF, Niu DK (2006). Replication-associated strand asymmetries in vertebrate genomes and implications for replicon size, DNA replication origin, and termination. Biochem Biophys Res Commun.

[B38] Berezney R, Dubey DD, Huberman JA (2000). Heterogeneity of eukaryotic replicons, replicon clusters, and replication foci. Chromosoma.

[B39] Nikolaou C, Almirantis Y (2005). A study on the correlation of nucleotide skews and the positioning of the origin of replication: different modes of replication in bacterial species. Nucl Acids Res.

[B40] Branco MR, Pombo A (2006). Intermingling of chromosome territories in interphase suggests role in translocations and transcription-dependent associations. PLoS Biol.

[B41] Cai S, Lee CC, Kohwi-Shigematsu T (2006). SATB1 packages densely looped, transcriptionally active chromatin for coordinated expression of cytokine genes. Nat Genet.

[B42] Girard-Reydet C, Gregoire D, Vassetzky Y, Mechali M (2004). DNA replication initiates at domains overlapping with nuclear matrix attachment regions in the xenopus and mouse c-myc promoter. Gene.

[B43] Koina E, Piper A (2005). An inactive X specific replication origin associated with a matrix attachment region in the human X linked HPRT gene. J Cell Biochem.

[B44] de Belle I, Cai S, Kohwi-Shigematsu T (1998). The genomic sequences bound to special AT-rich sequence-binding protein 1 (SATB1) in vivo in Jurkat T cells are tightly associated with the nuclear matrix at the bases of the chromatin loops. J Cell Biol.

[B45] Dickinson LA, Joh T, Kohwi Y, Kohwi-Shigematsu T (1992). A tissue-specific MAR/SAR DNA-binding protein with unusual binding site recognition. Cell.

[B46] Evans K, Ott S, Hansen A, Koentges G, Wernisch L (2007). A comparative study of S/MAR prediction tools. BMC Bioinformatics.

[B47] Barrett T, Troup DB, Wilhite SE, Ledoux P, Rudnev D, Evangelista C, Kim IF, Soboleva A, Tomashevsky M, Edgar R (2007). NCBI GEO: mining tens of millions of expression profiles-database and tools. Nucleic Acids Res.

[B48] Hubbard TJ, Aken BL, Beal K, Ballester B, Caccamo M, Chen Y, Clarke L, Coates G, Cunningham F, Cutts T (2007). Ensembl 2007. Nucleic Acids Res.

